# The Importance of Metabolic Syndrome Status for the Risk of Non-Viral Hepatocellular Carcinoma: A Nationwide Population-Based Study

**DOI:** 10.3389/fonc.2022.863352

**Published:** 2022-05-04

**Authors:** Yuri Cho, Eun Ju Cho, Jeong-Ju Yoo, Young Chang, Goh Eun Chung, In Young Choi, Sang-Hyun Park, Kyungdo Han, Yoon Jun Kim, Jung-Hwan Yoon, Dong Wook Shin, Su Jong Yu

**Affiliations:** ^1^ Department of Internal Medicine, Center for Liver and Pancreatobiliary Cancer, National Cancer Center, Goyang, South Korea; ^2^ Department of Internal Medicine and Liver Research Institute, Seoul National University College of Medicine, Seoul, South Korea; ^3^ Department of Gastroenterology and Hepatology, Soonchunhyang University Bucheon Hospital, Bucheon, South Korea; ^4^ Department of Gastroenterology and Hepatology, Soonchunhyang University Seoul Hospital, Seoul, South Korea; ^5^ Department of Internal Medicine, Healthcare Research Institute, Gangnam Healthcare Center, Seoul National University Hospital, Seoul, South Korea; ^6^ Total Healthcare Center, Kangbuk Samsung Hospital, Sungkyunkwan University School of Medicine, Seoul, South Korea; ^7^ Department of Biostatistics, College of Medicine, The Soongsil University, Seoul, South Korea; ^8^ Supportive Care Center/Department of Family Medicine, Samsung Medical Center, Sungkyunkwan University School of Medicine, Seoul, South Korea; ^9^ Department of Clinical Research Design and Evaluation, Samsung Advanced Institute for Health Science and Technology (SAIHST), Sungkyunkwan University, Seoul, South Korea; ^10^ Department of Digital Health, Samsung Advanced Institute for Health Science and Technology (SAIHST), Sungkyunkwan University, Seoul, South Korea

**Keywords:** hepatocellular carcinoma, epidemiology, nonalcoholic fatty liver disease, obesity, metabolic syndrome

## Abstract

The positive association between metabolic syndrome (MetS) and hepatocellular carcinoma (HCC) has been suggested. However, no studies have yet looked at how the risk of developing HCC varies with changes in MetS status. Therefore, we aimed to investigate the association between changes in MetS and subsequent HCC development. Data were obtained from the Korean National Health Insurance Service. In this study, 5,975,308 individuals who participated in health screenings both in 2009–2010 and 2011–2012 were included. Individuals with preexisting viral hepatitis, liver cirrhosis, or cancer diagnoses were excluded. Subjects were divided into four groups according to change in MetS status during the 2-year interval screening (from 2009 to 2011): sustained non-MetS, transition to MetS, transition to non-MetS, and sustained MetS. Cox regression analysis was used to examine the hazard ratios of HCC. The subjects were followed through December 31, 2018. During a median of 7.3 years of follow-up, 25,880 incident HCCs were identified. Compared to the sustained non-MetS group, age, sex, smoking, alcohol, regular exercise, and body mass index-adjusted hazard ratios (95% confidence interval) for HCC development were 1.01 (0.97–1.05) for the transition to MetS group, 1.05 (1.003–1.09) for the transition to non-MetS group, and 1.07 (1.03–1.10) for the sustained MetS group. Stratified analyses according to age, sex, smoking, alcohol intake, exercise, diabetes mellitus, hypertension, dyslipidemia, and chronic kidney disease showed similar results. A significantly increased HCC risk was observed in the sustained MetS and transition to non-MetS groups. The baseline status of MetS was associated with the risk of HCC development. Strategies to improve MetS, especially targeting insulin resistance, might prevent HCC development.

## Introduction

Worldwide, hepatocellular carcinoma (HCC) is the third most frequent malignancy (1), and the incidence of HCC has increased dramatically in the last two decades in many of the developed countries of the world (2). The etiologies of HCC include hepatitis B virus or hepatitis C virus infection and alcohol. However, 5%−30% of the HCC cases do not have an identifiable risk factor, which is called “cryptogenic HCC” (3). A trend toward the higher proportion of nonalcoholic fatty liver disease (NAFLD) patients with cryptogenic HCC has been reported (4). This suggests that increased proportion of risk factors associated with NAFLD may have contributed to the development of cryptogenic HCC (5).

The metabolic syndrome (MetS) is a cluster of metabolically related risk factors for cardiovascular disease (CVD) (6). NAFLD is also known as a feature of MetS (7). Recent experimental translational studies are available as an evidence that supports that the components of MetS including central obesity, dyslipidemia, and insulin resistance might be the important factors for HCC (8–10).

Recently, experts have suggested that “metabolic (dysfunction)-associated fatty liver disease (MAFLD)” might be a more appropriate term to describe fatty liver diseases associated with metabolic dysfunction (11). This novel term emphasizes the role of metabolic dysfunction on clinical outcome of patients with fatty liver disease, which may identify subjects at a higher risk of hepatic outcomes (12). Some epidemiologic studies have reported the positive association between MetS and HCC (13–16). However, the temporal and probably causative relation between MetS and HCC remains open to discussion.

We hypothesized that the temporal changes in MetS status might be the significant factor for HCC. Thus, this nationwide population-based study investigated whether the temporal changes in MetS status or the status of MetS components impact on the incidence of HCC.

## Materials and Methods

### Data Source

The Korean government has a single mandatory health insurance system that covers nearly 97% of South Koreans. The remaining 3% are covered by the Medical Aid program. The Korean National Health Insurance Services (NHIS) manages all administrative processes and reimburses medical providers and pharmacies based on their claims data. Korean NHIS is a mandatory social insurance that covers virtually all Koreans except for Medicaid beneficiaries in the lowest income bracket (approximately 3% of the population).

It conducts biennial health examinations for all Korean employees of any age and those aged 40 or older. This prevention program aims to detect and treat CVD-related health conditions including hypertension, diabetes, and dyslipidemia early to reduce the burden of CVD and offers subsequent educational counseling or treatment referral for participants with identified health problems. The examinations consist of anthropometric measurements, laboratory tests (lipid profiles, blood glucose, etc.), and questionnaires on lifestyle behaviors (smoking, alcohol consumption, and physical activity). Therefore, the Korean NHIS database includes health information from all Korean people (~50 million) based on eligibility (age, sex, place of residence, income level, etc.), medical utilization (diagnosis code, diagnostic and therapeutic procedures, prescription, medical expenses), and results from health examinations. This database has been widely used for various epidemiologic studies (17, 18).

### Ethics Statement

This study was approved by the institutional review board of Seoul National University Hospital (IRB No. E-1912-024-1085). Anonymized and de-identified information was used for analyses; therefore, informed consent was not required. The database is open to all researchers whose study protocols are approved by the official review committee. All the methods were performed in accordance with relevant guidelines and regulations.

### Study Population

**Figure 1:**
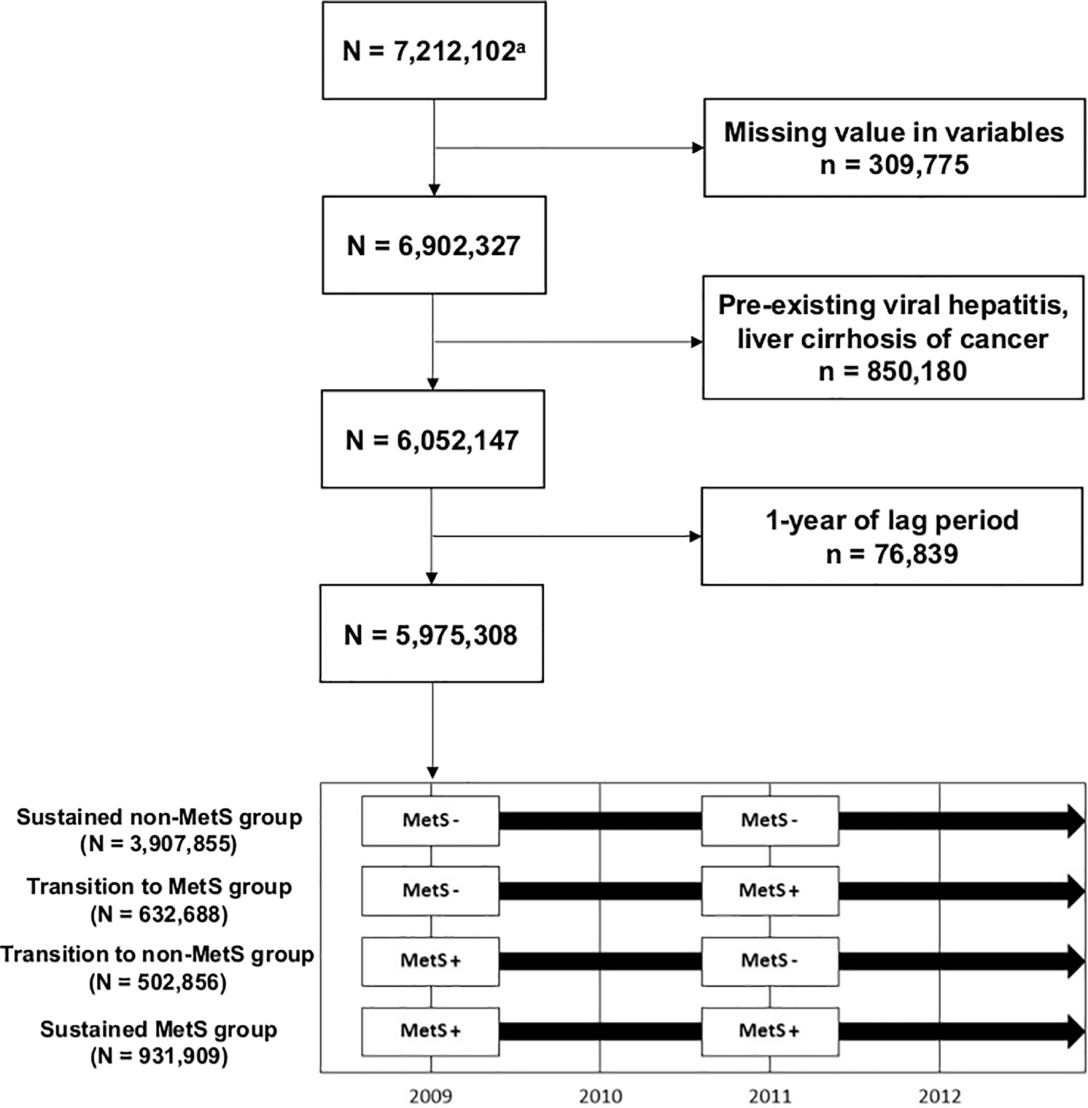
Flowchart of the study population selection process.

Among 7,212,102 subjects (age ≥20 years) who participated in health screenings both in 2009–2010 and 2011–2012, we excluded individuals with missing data (n = 309,775) and those with preexisting viral hepatitis [defined by International Classification of Disease version 10 (ICD-10) B15–B19], liver cirrhosis (LC, defined by ICD-10 K703, K746), or cancer diagnoses (n = 850,180). As subjects who developed HCC immediately after health examination may have an unclear temporal relationship with the MetS status identified at the health examination, we gave a 1-year lag time and further excluded 12,567 subjects diagnosed with HCC within a year after their second health examination. Therefore, 5,975,308 subjects were included in the final study population (**Figure 1**). The subjects were followed through December 31, 2018.

### Exposure Variable: Metabolic Syndrome Status

The definition of MetS followed the 2009 agreement of the International Diabetes Federation and American Heart Association/National Heart, Lung, and Blood Institute (6). By definition, the presence of three or more out of five risk factors constituted a MetS diagnosis: triglyceride (TG) ≥150 mg/dl or patient was taking lipid-lowering medication; high-density lipoprotein (HDL) <40 mg/dl in men and <50 mg/dl in women or use of lipid-lowering medication; systolic blood pressure ≥130 mmHg and/or diastolic blood pressure ≥85 mmHg or use of antihypertensive medication; fasting glucose ≥100 mg/dl or use of hypoglycemic agents; and abdominal obesity. Abdominal obesity was defined as waist circumference ≥90 cm for men and ≥85 cm for women according to the definition from the Korean Society for the Study of Obesity. (19) We compared 2009–2010 and 2011–2012 National Health Screening Program (NHSP) results. Using MetS change during biennial screening, we divided participants into four groups: sustained non-MetS, transition to MetS, transition to non-MetS, and sustained MetS.

### Outcome Variable: Hepatocellular Carcinoma

HCCs were identified using the following diagnoses from the ICD-10: Hepatocellular Carcinoma [C22.0]. In addition to ICD-10 codes, we confirmed cases of HCC through the registration program for critically ill or incurable diseases. Since 2005, the Korean government has provided co-payment reduction for registered cancer patients, and only patients whose cancer diagnoses were confirmed by physicians (after thorough evaluation) could be registered in this program.

### Covariates

Household income was classified into quartiles according to health insurance premium. Body mass index (BMI) was calculated as weight in kilograms divided by height in square meters. Waist circumference was measured at the midpoint between the lower margin of the ribs at the mid-axillary plane and the top of the iliac crest.

Health behavior, including smoking, alcohol consumption, and physical activity, was evaluated by self-reporting questionnaires. Smoking history was classified as never, former, and current smoker. Alcohol consumption was divided into three levels: none, mild-to-moderate (<30 g of alcohol/day), and heavy (≥30 g/day). Regular physical activity was defined as moderate physical activity for more than 30 min daily and more than 5 days per week over the past week. Income status was divided into quartiles based on the amount of health insurance premiums paid (Korean premiums are determined by income level), where those who received medical aid (the poorest 3%) were merged with the lowest income quartile.

Hypertension was defined as any of the following: systolic blood pressure ≥140 mmHg; diastolic blood pressure ≥90 mmHg; or treatment with an antihypertensive medication that was linked to the hypertension ICD-10 codes (I10–I13 and I15) and resulted in at least one claim in a year. Diabetes mellitus (DM) was defined as a blood glucose level ≥126 mg/dl or history of a hypoglycemic medication prescription that was linked to a diabetes ICD-10 code (E11–E14) and resulted in at least one claim in a year. Dyslipidemia was defined as total cholesterol ≥240 mg/dl or history of a lipid-lowering medication that was associated with an ICD-10 code (E78). Each medication code is described in **Supplementary Table S1**. Chronic kidney disease (CKD) was defined as an estimated glomerular filtration rate <60 ml/min per 1.73 m² of body surface area.

### Statistical Analysis

The comparison of baseline characteristics according to the change in MetS was conducted using independent *t*-tests for continuous variables and the chi-square test for categorical variables. The incidence rates of HCC were assessed as the incident cases divided by 1,000 person-years. Cox proportional hazards regression was performed to estimate the risk of HCC for the four MetS change groups (sustained non-MetS, transition to MetS, transition to non-MetS, and sustained MetS). Multivariable analyses were adjusted for age, sex, smoking history, alcohol consumption, physical activity, and BMI. Stratified analyses were performed according to age (<65 vs. ≥65 years old), sex (men vs. women), smoking, alcohol (average <30 g/day vs. heavy drinking ≥30 g/day), regular exercise (≥30 min of moderate physical activity ≥five times per week or ≥20 min of strenuous physical activity ≥three times per week). The statistical analyses were performed using SAS version 9.4 (SAS Institute Inc., Cary, NC, USA). In this study, *p*-values <0.05 were considered statistically significant.

## Results

### Characteristics of Study Subjects

A total of 5,975,308 subjects were enrolled in the study. Among the total subject group, 3,907,855 (65.4%) remained normal during the first and the second NHSP (sustained non-MetS group). Newly developed MetS was seen in 632,688 (10.6%) in the second screening (transition to MetS group), and 502,856 (8.4%) had MetS at the first screening that normalized at the second screening (transition to non-MetS group). Sustained MetS was noted for 931,909 (15.6%) during the two screenings (sustained MetS group).

All characteristics at 2011–2012 national health examinations were significantly different among the four groups (*p* < 0.001) (**Table 1**). Heavy drinking was higher in the transition to MetS group (9.3%), transition to non-MetS group (8.6%), and sustained MetS group (8.6%) than that in the sustained non-MetS group (6.6%). The regular exercise rate was higher in the transition to non-MetS group (22.3%) than in other groups. DM (32.0%), hypertension (66.0%), dyslipidemia (29.8%), and CKD (10.3%) were higher in the sustained MetS group than in other groups. The baseline characteristics of the study population at the first national health examinations are described in **Supplementary Table S2**.

**Table 1:** Baseline characteristics of the study population (at the time of second national health examinations).

	Change in the presence of MetS during 2 years					
	Sustained non-MetS group	Transition to MetS group	Transition to non-MetS group	Sustained MetS group	*p-*value	ASD
Number of subjects	3,907,855	632,688	502,856	931,909		
Age, years	45.4 ± 12.7	52.2 ± 13.0	52.8 ± 12.9	57.1 ± 12.5	<0.0001	0.51681
Men (%)	2,229,163 (57.0)	389,660 (61.6)	311,410 (61.9)	500,751 (53.7)	<0.0001	0.21245
Smoking status					<0.0001	0.16147
Never smoker (%)	2,293,421 (58.7)	341,236 (53.9)	272,991 (54.3)	553,657 (59.4)		
Ex-smoker (%)	627,632 (16.1)	121,891 (19.3)	97,188 (19.3)	169,092 (18.1)		
Current smoker (%)	986,802 (25.3)	169,561 (26.8)	132,677 (26.4)	209,160 (22.4)		
Alcohol consumption					<0.0001	0.13315
0 g/day (%)	1,866,282 (47.8)	316,308 (50.0)	257,227 (51.2)	537,408 (57.7)		
<30 g/day (%)	1,784,713 (45.7)	257,563 (40.7)	202,285 (40.2)	314, 829 (33.8)		
≥30 g/day (%)	256,860 (6.6)	58,817 (9.3)	43,344 (8.6)	79,672 (8.6)		
Regular physical activity (%)	780,532 (20.0)	124,932 (19.8)	112,037 (22.3)	189,783 (20.4)	<0.0001	0.06024
Body weight, kg	62.3 ± 10.6	68.5 ± 12.2	67.2 ± 11.8	69.9 ± 13.0	<0.0001	0.57862
BMI, kg/m^2^	22.8 ± 2.8	25.3 ± 3.0	24.9 ± 2.9	26.3 ± 3.2	<0.0001	0.84728
WC, cm	77.7 ± 8.0	85.5 ± 7.9	83.2 ± 7.6	88.0 ± 8.2	<0.0001	0.99818
SBP, mmHg	118.8 ± 13.3	129.9 ± 13.6	125.2 ± 13.9	131.6 ± 14.6	<0.0001	0.83427
DBP, mmHg	74.4 ± 9.2	80.7 ± 9.6	78.0 ± 9.4	80.9 ± 10.0	<0.0001	0.68391
Comorbidities						
Hypertension (%)	466,743 (11.9)	261,519 (41.3)	168,826 (33.6)	614,732 (66.0)	<0.0001	1.386
DM (%)	103,901 (2.7)	79,209 (12.5)	52,487 (10.4)	298,027 (32.0)	<0.0001	0.85838
Dyslipidemia (%)	342,374 (8.8)	193,668 (30.6)	77,187 (15.4)	371,218 (39.8)	<0.0001	0.99773
Chronic kidney disease (%)	485,762 (3.9)	40,362 (6.4)	31,427 (6.3)	95,557 (10.3)	<0.0001	0.25767
Laboratory results						
Fasting glucose (mg/dl)	92.1 ± 14.8	104.8 ± 23.9	98.8 ± 23.5	113.7 ± 34.0	<0.0001	0.64994
Total cholesterol (mg/dl)	192.3 ± 33.7	205.1 ± 40.3	201.5 ± 37.0	200.6 ± 42.6	<0.0001	0.24726
Triglycerides (mg/dl)	93 (67–130)	171 (122–227)	122 (92–163)	177 (124–247)	<0.0001	0.91686
HDL cholesterol (mg/dl)	58.0 ± 19.3	49.6 ± 24.0	53.0 ± 30.0	48.1 ± 19.2	<0.0001	0.52733
Creatinine (mg/dl)	.03 ± 1.2	1.04 ± 1.1	1.03 ± 1.1	1.01 ± 1.1	<0.0001	0.02064
ALT (IU/L)	18 (14–25)	25 (18–35)	22 (16–31)	25 (18–37)	<0.0001	0.443
AST (IU/L)	21 (18–26)	24 (20–30)	23 (19–29)	25 (20–31)	<0.0001	0.3
GGT (IU/L)	20 (14–32)	31 (20–54)	28 (19–47)	33 (21–58)	<0.0001	0.379

MetS, metabolic syndrome; HCC, hepatocellular carcinoma; BMI, body mass index; WC, waist circumference; SBP, systolic blood pressure; DBP, diastolic blood pressure; DM, diabetes mellitus; HDL, high-density lipoprotein; ALT, alanine aminotransferase; AST, aspartate aminotransferase; GGT, gamma-glutamyl transferase; ASD, absolute standardized difference.

Values are presented as mean ± standard deviation or median (range) for continuous variables and number (%) for categorical variables.

### Incidence of Hepatocellular Carcinoma According to Baseline Metabolic Syndrome and Components

Median follow-up duration of the study population was 7.3 years. The HCC incidence rate for the non-MetS group was 0.59 cases per 1,000 person-years and 0.95 cases in the MetS group (**Table 2**). HCC development risk was higher in the MetS group [adjusted hazard ratio (aHR), 1.03; 95% confidence interval (CI), 1.004–1.06]. Without adjustment, all MetS components also showed higher risk of HCC development. After adjustment for age, sex, smoking, alcohol, regular exercise, and BMI, waist circumference (aHR 1.12, 95% CI 1.08–1.15), fasting glucose (aHR 1.17, 95% CI 1.14–1.20), and the presence of hypertension (aHR 1.09, 95% CI 1.06–1.12) were the significant factors for developing HCC. However, low HDL (aHR 0.96, 95% CI 0.96–0.99) and high TG (aHR 0.83, 95% CI 0.81–0.85) were inversely associated with HCC risk.

**Table 2:** Incidence of HCC according to the components of metabolic syndrome.

	No. of subjects	HCC cases (n)	Incidence of HCC (1,000 person-years)	Crude HR (95% CI)	Adjusted HRModel 1^b^(95% CI)	Adjusted HRModel 2^c^(95% CI)
**Metabolic syndrome** ^a^						
No	4,410,711	16,508	0.591	1 (reference)	1 (reference)	1 (reference)
Yes	1,564,597	9,372	0.950	1.61 (1.57–1.65)	1.12 (1.09–1.15)	1.03(1.00–1.06)
*p*-value				<0.0001	<0.0001	0.0246
**By metabolic syndrome components**						
Waist circumference						
No	4,368,462	16,986	0.615	1 (reference)	1 (reference)	1 (reference)
Yes	1,606,846	8,894	0.875	1.42 (1.39–1.46)	1.21 (1.17–1.24)	1.12 (1.08–1.15)
*p*-value				<0.0001	<0.0001	<0.0001
Fasting glucose						
No	4,078,226	14,532	0.562	1 (reference)	1 (reference)	1 (reference)
Yes	1,897,082	11,348	0.951	1.69 (1.65–1.74)	1.21 (1.18–1.24)	1.17 (1.14–1.2)
*p*-value				<0.0001	<0.0001	<0.0001
HDL cholesterol						
No	4,514,420	18,743	0.656	1 (reference)	1 (reference)	1 (reference)
Yes	1,460,888	7,137	0.772	1.17 (1.14–1.21)	0.98 (0.95–1.01)	0.96 (0.93–0.99)
*p*-value				<0.0001	0.1151	0.0031
Blood pressure						
No	3,256,666	10,210	0.494	1 (reference)	1 (reference)	1 (reference)
Yes	2,718,642	15,670	0.915	1.85 (1.81–1.9)	1.15 (1.12–1.18)	1.09 (1.06–1.12)
*p*-value				<0.0001	<0.0001	<0.0001
Triglycerides						
No	3,905,281	16,409	0.664	1 (reference)	1 (reference)	1 (reference)
Yes	2,070,027	9,471	0.724	1.09 (1.06–1.12)	0.90 (0.88–0.92)	0.83 (0.81–0.85)
*p*-value				<0.0001	<0.0001	<0.0001

HCC, hepatocellular carcinoma; HDL, high-density lipoprotein; HR, hazard ratio; CI, confidence interval; BMI, body mass index; DM, diabetes mellitus.

a Metabolic syndrome and components were defined from blood tests and anthropometric measurements from the 2009−2010 examinations: waist circumference ≥90 cm (men) or 85 cm (women), systolic blood pressure ≥130 mmHg and/or diastolic blood pressure ≥85 mmHg, fasting glucose ≥100 mg/dl, triglycerides ≥150 mg/dl, HDL <40 mg/dl (men) or 50 mg/dl (women). The presence of three or more out of five components was regarded as metabolic syndrome.

b Model 1: adjusted for age and sex.

c Model 2: adjusted age, sex, smoking, alcohol, regular exercise, and BMI.

### Incidence of Hepatocellular Carcinoma According to Change in Metabolic Syndrome and Components

Compared to the sustained non-MetS group, other groups showed a higher risk of HCC development. The risk for HCC did not significantly increase for transition to MetS group (aHR 1.01; 95% CI, 0.97–1.05) compared with sustained non-MetS group. However, the risk for HCC was significantly increased among both transition to non-MetS (aHR 1.05; 95% CI, 1.003–1.09) and sustained MetS group (aHR 1.07; 95% CI, 1.03–1.10).

By individual components, those who developed high waist circumference (aHR 1.06; 95% CI, 1.01–1.11) and fasting glucose (aHR 1.08; 95% CI, 1.05–1.13) showed slightly increased risk for HCC compared to sustained non-MetS group. Those who showed sustained high waist circumference (aHR 1.21, 95% CI 1.16–1.26), fasting glucose (aHR 1.25, 95% CI 1.21–1.28), and blood pressure (aHR 1.12, 95% CI 1.09–1.16) were associated with higher HCC risk compared to sustained non-MetS group. In comparison, those who showed normalization of waist circumference (aHR 1.12, 95% CI 1.07–1.18 vs. aHR 1.21, 95% CI 1.16–1.26), blood pressure (aHR 1.02, 95% CI 0.97–1.06 vs. aHR 1.12, 95% CI 1.09–1.16), and fasting glucose (aHR 1.06, 95% CI 1.01–1.10 vs. aHR 1.25, 95% CI 1.21–1.28) showed an elevated risk for HCC compared to sustained non-MetS group, but the risks were lower than those in the sustained high group. For HDL cholesterol, sustained low HDL cholesterol group showed a slightly lower risk than that of sustained non-MetS group (aHR 0.95, 95% CI 0.91–0.98). For TG, all of transition to normal (aHR 0.87, 95% CI 0.84–0.90), transition to high TG (aHR 0.90, 95% CI 0.87–0.94), and sustained high TG group (aHR 0.78, 95% CI 0.75–0.80) showed a lower risk of HCC than the sustained normal TG group (**Table 3**). When we changed the reference group as sustained MetS group, the sustained non-MetS group (aHR 0.94, 95% CI 0.91–0.97) showed a lower risk of HCC incidence compared with that of the sustained MetS group (**Supplementary Table S3**).

**Table 3:** Incidence of HCC according to metabolic change during 2 years of follow-up.

	Number of subjects	Number of HCC cases	Incidence of HCC (1,000 person-years)	Crude HR(95% CI)	Adjusted HRModel 1^b^(95% CI)	Adjusted HRModel 2^c^(95% CI)
MetS^a^ status						
Sustained non-MetS group	3,907,855	13,776	0.556	1 (reference)	1 (reference)	1 (reference)
Transition to MetS group	632,688	3,270	0.818	1.47 (1.42–1.53)	1.08 (1.04–1.12)	1.01 (0.97–1.05)
Transition to non-MetS group	502,856	2,732	0.861	1.55 (1.49–1.61)	1.11 (1.06–1.15)	1.05 (1.00–1.09)
Sustained MetS group	931,909	6,102	1.040	1.87 (1.81–1.92)	1.18 (1.14–1.21)	1.07 (1.03–1.10)
*p*-value				<0.0001	<0.0001	0.0018
**By changes of MetS components**						
Waist circumference						
No → No	3,923,615	14,681	0.591	1 (reference)	1 (reference)	1 (reference)
No → Yes	513,925	2,317	0.713	1.20 (1.15–1.26)	1.1 (1.05–1.15)	1.06 (1.01–1.11)
Yes → No	444,847	2,305	0.821	1.39 (1.33–1.45)	1.15 (1.10–1.21)	1.12 (1.07–1.18)
Yes → Yes	1,092,921	6,577	0.951	1.60 (1.56–1.65)	1.29(1.251,1.33)	1.21 (1.16–1.26)
*p*-value				<0.0001	<0.0001	<0.0001
Fasting glucose						
No → No	3,359,245	11,326	0.531	1 (reference)	1 (reference)	1 (reference)
No → Yes	798,529	3,754	0.744	1.40 (1.35–1.45)	1.12 (1.08–1.16)	1.08 (1.05–1.13)
Yes → No	718,981	3,206	0.705	1.33 (1.28–1.38)	1.08 (1.04–1.12)	1.06 (1.01–1.10)
Yes → Yes	1,098,553	7,594	1.103	2.08 (2.02–2.14)	1.30 (1.26–1.34)	1.25 (1.21–1.28)
*p*-value				<0.0001	<0.0001	<0.0001
HDL-cholesterol						
No → No	3,906,588	15,915	0.644	1 (reference)	1 (reference)	1 (reference)
No → Yes	672,699	3,250	0.764	1.18 (1.14–1.23)	0.99 (0.96–1.03)	0.98 (0.94–1.01)
Yes → No	607,832	2,828	0.736	1.14 (1.10–1.19)	1.02 (0.98–1.06)	1.01 (0.97–1.05)
Yes → Yes	788,189	3,887	0.779	1.21 (1.17–1.25)	0.97 (0.94–1.01)	0.95 (0.91–0.98)
*p*-value				<0.0001	0.2159	0.0143
Blood pressure						
No → No	2,599,455	7,596	0.460	1 (reference)	1 (reference)	1 (reference)
No → Yes	774,397	3,179	0.649	1.41 (1.35–1.47)	1.06 (1.02–1.10)	1.01 (0.97–1.06)
Yes → No	657,211	2,614	0.628	1.37 (1.31–1.43)	1.05 (1.00–1.10)	1.02 (0.97–1.06)
Yes → Yes	1,944,245	12,491	1.022	2.22 (2.16–2.29)	1.20 (1.16–1.23)	1.12 (1.09–1.16)
*p*-value				<0.0001	<0.0001	<0.0001
Triglycerides						
No → No	3,241,284	13,171	0.642	1 (reference)	1 (reference)	1 (reference)
No → Yes	773,869	3,508	0.716	1.12 (1.07–1.16)	0.93 (0.89–0.96)	0.87 (0.84–0.9)
Yes → No	663,997	3,238	0.772	1.20 (1.16–1.25)	0.96 (0.92–0.99)	0.90 (0.87–0.94)
Yes → Yes	1,296,158	5,963	0.728	1.13 (1.1–1.17)	0.87 (0.84–0.90)	0.78 (0.75–0.80)
*p*-value				<0.0001	<0.0001	<0.0001

HCC, hepatocellular carcinoma; HDL, high-density lipoprotein; HR, hazard ratio; CI, confidence interval; BMI, body mass index; DM, diabetes mellitus.

a Metabolic syndrome and components were defined from blood tests and anthropometric measurements from the 2009−2010 examinations: waist circumference ≥90 cm (men) or 85 cm (women), systolic blood pressure ≥130 mmHg and/or diastolic blood pressure ≥85 mmHg, fasting glucose ≥100 mg/dl, triglycerides ≥150 mg/dl, HDL <40 mg/dl (men) or 50 mg/dl (women). The presence of three or more out of five components was regarded as metabolic syndrome.

b Model 1: adjusted for age and sex.

c Model 2: adjusted age, sex, smoking, alcohol, regular exercise, and BMI.

### Stratified Analyses

**Figure 2:**
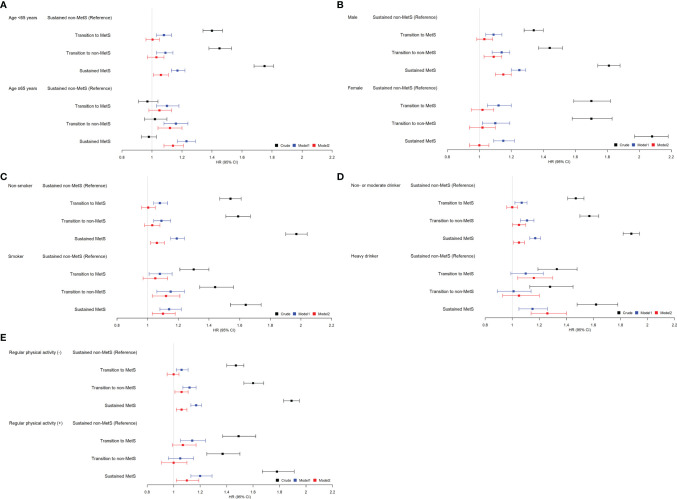
Relationship between the change in metabolic syndrome (MetS) status and hepatocellular carcinoma (HCC) incidence stratified by patient characteristics. **(A)** Age, **(B)** sex, **(C)** smoking, **(D)** alcohol, **(E)** regular physical activity. *Adjusted for age, sex, smoking, alcohol, regular exercise, and body mass index (BMI).

We performed stratified analyses according to age (<65 vs. ≥65 years), sex (men vs. women), smoking, alcohol (<30 vs. ≥30 g/day), and regular exercise (**Figure 2**). Stratified analyses also showed generally similar associations between the change in MetS and with the risk of HCC development (**Supplementary Table S4**). However, the association between sustained MetS and HCC risk was larger in older person (aHR 1.14, 95% CI 1.08–1.21 vs. aHR 1.06, 95% CI 1.01–1.11 in younger person), men (aHR 1.15, 95% CI 1.10–1.2 vs. aHR 1.00, 95% CI 0.94–1.06 in women), and heavy drinker (aHR 1.26, 95% CI 1.14–1.40 vs. aHR 1.05, 95% CI 1.01–1.09 in non- or moderate drinker).

We also additionally performed stratified analyses according to age subgroups (<45, 45–55, >55 years). Interestingly, among subjects with <45 and 45–55 years old, there is no significant difference in HRs for the incidence of HCC among the four MetS change groups (**Supplementary Table S5**). Only among subjects >55 years old, the sustained MetS group showed a higher risk for HCC incidence compared to that of the sustained non-MetS group (aHR 1.18, 95% CI 1.1–1.26).

## Discussion

To the best of our knowledge, this is the first population-based study that examined the HCC risks according to MetS state changes. The uniqueness of our study is that we measured MetS status twice with 2-year interval, thus enabling estimating the HCC risk by the changes of metabolic status. We found that MetS was significantly associated with the risk of HCC, and the risk was greater when the MetS was sustained. Among the components of MetS, central obesity, impaired fasting glucose, and high blood pressure were associated with an increased risk of HCC. However, the risk of HCC development was lower in people who returned to non-MetS status than that of those who had sustained MetS status.

Our study is consistent with previous studies that show a positive association between MetS and the HCC risk. A previous prospective European cohort study reported that MetS increases the risk of HCC with adjusted relative risk of 1.35 (95% CI 1.12−1.61) (15). A population-based case-control study in the United States also showed that MetS increases the risk of HCC, with odds ratio of 2.13 (95% CI 1.96−2.31) (14). Those studies reported a slightly higher risk of HCC than that in our study probably due to including patients with underlying chronic liver disease (e.g., chronic viral hepatitis, alcohol-related liver disease). In addition, we showed that sustained MetS was associated with 7% higher HCC risk, further suggesting a causal association between MetS and HCC. The baseline status of MetS increased HCC risk by 3%, which is lower than that of sustained MetS group. The status of MetS can change dynamically, (20) so one time measurement would not be optimal. A previous cohort study using data from the Korean National Insurance Service from 2002 failed to observe a significantly increased risk of liver cancer among individuals with a high-risk metabolic profile (aHR 0.93; 95% CI, 0.75–1.16 for men, aHR 1.18; 95% CI, 0.78–1.77 for women) (21). Interestingly, our study including individuals who participated in health screenings both in 2009–2010 and 2011–2012 is the first to show an increased HCC risk among individuals with sustained MetS, which might reflect the temporal changes of HCC epidemiology in Korea.

This study’s uniqueness is that we investigated the impact of MetS temporal changes on HCC development. Our data show that if subjects have had MetS even once, the risk of HCC increased. Actually, the mean age of non-viral causes of HCC is higher than that of viral causes of HCC, (22) which is one of the pieces of evidence that a long exposure period of MetS influences the incidence of HCC (23). However, when we look at the risk by changes of individual component, transition to central obesity (aHR 1.06), impaired fasting glucose (aHR 1.08) showed significant elevation of HCC risk. We expect that the risk of HCC in this transition to MetS group might increase significantly in the future long-term follow-up study, as other previous studies have reported (24, 25). The results from our study also support that the at-risk population for HCC may be far larger than estimated and highlight the need defining appropriate strategies for HCC risk stratification among those with MetS and early detection in individuals without LC.

The incidence of MetS and HCC continues to increase worldwide (25). Previous studies have suggested that there is a link between the increase in HCC and MetS (26, 27). MetS might promote HCC development in multiple ways. Insulin resistance leads to an increase in insulin-like growth factor (IGF)-1, the most powerful activator of cellular proliferation including cancer cells (28). The aberrant activation of IGF signaling pathways is an important mechanism in the development of HCC. IGF-1 and IGF-2 interact with specific receptors. IGF-2 may also act in early hepatocarcinogenesis (29). Another key mechanism involves peroxisome proliferator-activated receptor (PPAR)-γ that is most highly expressed in white or brown adipose tissue and fatty liver. The inhibitory action of PPAR-γ on hepatocarcinogenesis *via* the upregulation of plasminogen activator factor 1 was reported (30). MetS also increases several adipokines, hormone levels, and the signaling pathways that create the ideal tumor microenvironment for developing steatosis and hepatic inflammation (31). Adipokines, such as leptin, may mediate HCC development through their effects on angiogenesis (32). Furthermore, MetS may lead to a state of chronic inflammation. Excess consumption of fatty acids and glucose can lead to the increased expression of several signaling molecules with known importance in carcinogenesis, invasion, and metastasis, including nuclear factor-κB, epidermal growth factor, and fibroblast growth factor (33).

Our data show that the risk of HCC development in this transition to non-MetS group was lower in the sustained MetS group, suggesting that there might be a preventive effect for HCC development by improving MetS status. The difference in relative risk between transition to non-MetS group and sustained MetS group was even more prominent when compared by individual components, e.g., waist circumference (aHR 1.12 vs. 1.21), fasting glucose (1.06 vs. 1.25), and blood pressure (1.02 vs. 1.12). This suggests the need to reverse MetS to prevent the risk of HCC. Efforts should start focusing at controlling MetS components such as DM, hypertension, dyslipidemia, and obesity (34). Weight control losing >5%–7% (35) is the key factor in managing MetS with a combination of reduced total caloric intake more than 500 kcal per day and increased physical activity (36). Pharmacological hypoglycemic treatments including PPAR-γ agonists (e.g., pioglitazone) (37), metformin (38), or glucagon-like peptide-1 agonists [e.g., liraglutide (39) or semaglutide (40)] have shown histological improvement in nonalcoholic steatohepatitis (NASH) patients. Also, well-established pharmacological treatments for hypertension and dyslipidemia, such as statins, have shown a positive preventive effect on the progression of cirrhosis and HCC development. A multidimensional approach for managing MetS might reduce HCC risk.

Interestingly, high TG levels were inversely associated with HCC risk in this study, which is consistent with our previous report (41). Dysregulation of cholesterol metabolism itself might act as a part of hepatocarcinogenesis. Cholesterol is involved in numerous biochemical pathways that are potentially relevant in HCC development, including several cytokine and signaling pathways. Interleukin-6, tumor necrosis factor-α, and interleukin-1 inhibit TG synthesis. These proinflammatory cytokines could act as carcinogens or cofactors for hypocholesterolemia and hepatocarcinogenesis.

Subjects with hypertension showed increased risk of HCC development. Essential hypertension is associated with MetS, which is characterized by insulin resistance and is strongly correlated with the development of NAFLD (42). Essential hypertension has been associated with an increased HCC mortality but the mechanisms of this positive relationship are not well known. The effects of high blood pressure might influence the progression of hepatic fibrosis. In animal models, hypertension was a potential risk factor for liver injury through glucose intolerance and decreased anti-inflammatory mechanism (43). Recently, Feng et al. reported that renin-angiotensin inhibitor was associated with longer time-to-recurrence and overall survival of HCC patients with essential hypertension after hepatectomy (44). Arterial hypertension might play a fundamental role in liver injury and progression of hepatic fibrosis leading to increased risk of HCC development.

Several limitations of this study must be acknowledged. Importantly, in this study, there is the chance that cirrhosis is not identified by Korean NHIS. Different definitions of cirrhosis might carry essentially misclassification bias. In real-life clinical practice, physicians use different methods to diagnose cirrhosis, which may affect coding the diagnosis in the medical record system. Liver biopsy is not a routine clinical practice, and liver stiffness measurement is not always feasible. It is possible that some of those who had unrecognized cirrhosis due to radiological ambiguity of the diagnosis of cirrhosis were included in this analysis. Second, the time period analyzed during our study is relatively short. Regarding that patients with NASH and fibrosis can progress to cirrhosis from 0% at 5 years to 12% over 8 years, (45) it is difficult to determine how the duration of risk factor exposure influences the development of HCC. However, it is reported that a third of NASH-related HCC develops in a non-cirrhotic liver, (46) which might lead to the positive findings of this study. Third, as with all epidemiological studies, our study cannot establish a causal relationship. Fourth, subjects with MetS (especially sustained MetS group) were significantly older than others. However, age-matching analysis was hard to apply in our 4-group analyses. Instead, we performed stratified analysis according to age group to minimize the confounding effect of age. Only among subjects >55 years old, sustained MetS group showed a higher risk for HCC incidence compared to that of the sustained non-MetS group. This might be due to the difference in exposure time to MetS. The effect of MetS status on hepatocarcinogenesis might be more prominent in older subjects (>55 years) who have been exposed to MetS for a relatively longer period of time than younger subjects (<55 years). Regarding that NAFLD-related HCC tends to occur in older individuals than viral hepatitis-related HCC (47), the result of this study is consistent with those of previous reports (48, 49).

In conclusion, this study demonstrated that MetS was significantly associated with the risk of HCC, and the risk was greater when MetS was sustained. Among the components of MetS, central obesity, impaired fasting glucose, and high blood pressure were associated with increased risk of HCC, suggesting the role of insulin resistance in the development of HCC. However, the risk of HCC development in the transition to non-MetS group was lower than that of the sustained MetS group, suggesting that there might be a preventive effect for HCC development by improving MetS status. Strategies to improve MetS, especially targeting insulin resistance, might help to prevent HCC development.

## Data Availability Statement

The datasets presented in this study can be found in online repositories. The names of the repository/repositories and accession number(s) can be found in the article/**Supplementary Material**.

## Ethics Statement

The studies involving human participants were reviewed and approved by the institutional review board of Seoul National University Hospital (IRB No. E-1912-024-1085). The ethics committee waived the requirement of written informed consent for participation.

## Author Contributions

SJY and DWS have full access to all the data in the study and take responsibility for the integrity of the data and the accuracy of the data analysis. YuC, EJC, JJY, YoC, GEC, and IYC collected data. YuC, EJC, SHP, KH, SJY, and DWS analyzed and interpreted data. YuC, SJY, and DWS wrote the article. All authors contributed to the interpretation and discussion of the results and read and approved the final article.

## Funding

This work was supported by grants from the Seoul National University Hospital Research Fund (06-2020-4150), Liver Research Foundation of Korea as part of the Bio Future Strategies Research Project, and the National Research Foundation of Korea grant funded by the Korea government (2021R1A2C4001401).

## Conflict of Interest

The authors declare that the research was conducted in the absence of any commercial or financial relationships that could be construed as a potential conflict of interest.

## Publisher’s Note

All claims expressed in this article are solely those of the authors and do not necessarily represent those of their affiliated organizations, or those of the publisher, the editors and the reviewers. Any product that may be evaluated in this article, or claim that may be made by its manufacturer, is not guaranteed or endorsed by the publisher.
