# Supplementary material for: The Importance of Metabolic Syndrome Status for the Risk of Non-Viral Hepatocellular Carcinoma: A Nationwide Population-Based Study
Source: Front Oncol. 2022 May 4;12:863352. doi: 10.3389/fonc.2022.863352 (PMC9116136; doi:10.3389/fonc.2022.863352)
Supplement: Supplementary file 1 [file DataSheet_1.docx]

Supplementary Material

# Supplementary Tables

**Supplementary Table 1. Medication codes**

|  | ATC codes |
| --- | --- |
| **Hypoglycemic agent** |  |
| acarbose | A10BF01 |
| albiglutide | A10BJ04 |
| alogliptin | A10BH04 |
| dapagliflozin | A10BK01 |
| dulaglutide | A10BJ05 |
| empagliflozin | A10BK03 |
| ertugliflozin | A10BK04 |
| exenatide | A10BJ01 |
| gemigliptin | A10BH06 |
| glibenclamide | A10BB01 |
| gliclazide | A10BB09 |
| glimepiride | A10BB12 |
| glipizide | A10BB07 |
| gliquidone | A10BB08 |
| human insulin | A10A |
| linagliptin | A10BH05 |
| lixisenatide | A10BJ03 |
| lobeglitazone | A10BG04 |
| metformin | A10BA02 |
| miglitol | A10BF02 |
| nateglinide | A10BX03 |
| pioglitazone | A10BG03 |
| repaglinide | A10BX02 |
| rosiglitazone | A10BG02 |
| saxagliptin | A10BH03 |
| sitagliptin | A10BH01 |
| teneligliptin | A10BH08 |
| vildagliptin | A10BH02 |
| voglibose | A10BF03 |
| acarbose | A10BF01 |
| albiglutide | A10BJ04 |
| alogliptin | A10BH04 |
| dapagliflozin | A10BK01 |
| dulaglutide | A10BJ05 |
| empagliflozin | A10BK03 |
| **Antihypertensive medication** |  |
| amlodipine | C08CA01 |
| atenolol | C07AB03 |
| barnidipine | C08CA12 |
| benazepril | C09AA07 |
| benidipine | C08CA15 |
| betaxolol | C07AB05 |
| bevantolol | C07AB06 |
| bisoprolol | C07AB07 |
| cadralazine | C02DB04 |
| candesartan cilexetil | C09CA06 |
| captopril | C09AA01 |
| carvedilol | C07AG02 |
| cicletanine | C03BX03 |
| cilazapril | C09AA08 |
| cilnidipine | C08CA14 |
| delapril | C09AA12 |
| enalapril maleate | C09AA02 |
| eprosartan mesylate | C09CA02 |
| felodipine | C08CA02 |
| fosinopril | C09AA09 |
| hydralazine | C02DB02 |
| hydrochlorothiazide | C03AA03 |
| imidapril | C09AA16 |
| indapamide | C03BA11 |
| irbesartan | C09CA04 |
| isradipine | C08CA03 |
| labetalol | C07AG01 |
| lacidipine | C08CA09 |
| lercanidipine | C08CA13 |
| lisinopril | C09AA03 |
| losartan potassium | C09CA01 |
| manidipine | C08CA11 |
| metoprolol succinate | C07AB02 |
| minoxidil | C02DC01 |
| moexipril | C09AA13 |
| moxonidine | C02AC05 |
| nebivolol | C07AB12 |
| nicardipine | C08CA04 |
| nilvadipine | C08CA10 |
| nitrendipine | C08CA08 |
| olmesartan | C09CA08 |
| perindopril | C09AA04 |
| Phenoxybenzamine | C04AX02 |
| prazosin | C02CA01 |
| quinapril | C09AA06 |
| ramipril | C09AA05 |
| spironolactone | C03DA01 |
| telmisartan | C09CA07 |
| temocapril | C09AA14 |
| **Lipid lowering medication** |  |
| simvastatin | C10AA01 |
| lovastatin | C10AA02 |
| fluvastatin | C10AA04 |
| atorvastatin | C10AA05 |
| rosuvastatin | C10AA07 |
| pitavastatin | C10AA08 |
| bezafibrate | C10AB02 |
| gemfibrozil | C10AB04 |
| fenofibrate | C10AB05 |
| ciprofibrate | C10AB08 |
| etofibrate | C10AB09 |
| ezetimibe | C10AX09 |
| simvastatin | C10AA01 |
| lovastatin | C10AA02 |
| fluvastatin | C10AA04 |
| atorvastatin | C10AA05 |

**Supplementary Table 2. Baseline characteristics of the study population at 2 years before from the baseline (2009–2010 national health examinations)**

|  | Change in the presence of metabolic syndrome during 2 years | | | | |
| --- | --- | --- | --- | --- | --- |
|  | No → No | No →Yes | Yes → No | Yes → Yes | *p-*value |
| n | 3,907,855 | 632,688 | 502,856 | 931,909 |  |
| Age, years | 43.4 ± 12.7 | 50.2 ± 13.0 | 50.8 ± 12.9 | 55.1 ± 12.5 | <0.0001 |
| Male (%) | 2,229,163 (57.0) | 389,660 (61.6) | 311,410 (61.9) | 500,751 (53.7) | <0.0001 |
| Smoking status |  |  |  |  | <0.0001 |
| Never-smoker (%) | 2,301,854 (58.9) | 342,536 (54.1) | 274,620 (54.6) | 555,648 (59.6) |  |
| Ex-smoker (%) | 566,236 (14.5) | 103,611 (16.4) | 90,146 (17.9) | 152,539 (16.4) |  |
| Current smoker (%) | 1,039,765 (26.6) | 186,541 (29.5) | 138,090 (27.5) | 223,722 (24.0) |  |
| Alcohol consumption |  |  |  |  | <0.0001 |
| 0 g/day (%) | 1,869,729 (47.9) | 313,789 (49.6) | 253,826 (50.5) | 529,023 (56.8) |  |
| <30 g/day (%) | 1,766,562 (45.2) | 257,585 (40.7) | 200,679 (39.9) | 315,903 (33.9) |  |
| ≥30 g/day (%) | 271,564 (7.0) | 61,314 (9.7) | 48,351 (9.6) | 86,983 (9.3) |  |
| Regular physical activity (%) | 711,761 (18.2) | 127,323 (20.1) | 94,936 (18.9) | 182,300 (19.6) | <0.0001 |
| Body weight, kg | 62.0 ± 10.4 | 67.4 ± 11.6 | 68.2 ± 11.8 | 70.0 ± 12.7 | <0.0001 |
| BMI, kg/m^2^ | 22.7 ± 2.7 | 24.9 ± 2.8 | 25.2 ± 4.5 | 26.4 ± 4.3 | <0.0001 |
| WC, cm | 77.3 ± 8.1 | 83.2 ± 7.7 | 85.3 ± 8.6 | 88.0 ± 8.5 | <0.0001 |
| SBP, mmHg | 118.5 ± 13.2 | 125.0 ± 14.1 | 130.2 ± 13.6 | 132.1 ± 14.8 | <0.0001 |
| DBP, mmHg | 74.3 ± 9.2 | 78.1 ± 9.5 | 80.9 ± 9.6 | 81.6 ± 10.1 | <0.0001 |
| Comorbidities |  |  |  |  |  |
| Hypertension (%) | 419,958 (10.8) | 187,784 (29.7) | 194,318 (38.6) | 580,159 (62.3) | <0.0001 |
| DM (%) | 94,710 (2.4) | 49,985 (7.9) | 61,823 (12.3) | 259,137 (27.8) | <0.0001 |
| Dyslipidemia (%) | 309,074 (7.9) | 107,062 (16.9) | 118,634 (23.6) | 336,659 (36.1) | <0.0001 |
| Chronic kidney disease (%) | 212,219 (5.4) | 46,121 (7.3) | 41,580 (8.3) | 104,651 (11.2) | <0.0001 |
| Laboratory results |  |  |  |  |  |
| Fasting glucose, mg/dL | 91.6 ± 15.0 | 97.2 ± 22.5 | 105.1 ± 24.9 | 112.5 ± 33.7 | <0.0001 |
| Total cholesterol, mg/dL | 190.7 ± 36.6 | 204.8 ± 41.1 | 204.5 ± 46.9 | 206.3 ± 47.6 | <0.0001 |
| TG, mg/dL | 92 (66–128) | 125 (94–169) | 173 (128–227) | 182 (131–253) | <0.0001 |
| HDL-C, mg/dL | 59.0 ± 32.1 | 55.2 ± 33.6 | 49.9 ± 31.8 | 49.5 ± 32.4 | <0.0001 |
| Creatinine, mg/dL | 1.2 ± 1.5 | 1.1 ± 1.4 | 1.2 ± 1.5 | 1.1 ± 1.4 | <0.0001 |

**Abbreviations:** HCC, hepatocellular carcinoma; BMI, body mass index; WC, waist circumference; SBP, systolic blood pressure; DBP, diastolic blood pressure; DM, diabetes mellitus; TG, triglyceride; HDL-C, high-density lipoprotein cholesterol.

Values are presented as mean ± standard deviation or median (range) for continuous variables and number (%) for categorical variables.

**Supplementary Table 3. Incidence of HCC according to metabolic change during 2 years of follow-up with the reference group as sustained MetS group**

|  | Number of subjects | Number of HCC cases | Incidence of HCC | Crude HR | *P* value | Adjusted HR | *P* value | Adjusted HR | *P* value |
| --- | --- | --- | --- | --- | --- | --- | --- | --- | --- |
|  |  |  | (1,000 person-years) | (95% CI) |  | Model 1^b^ |  | Model 2^c^ |  |
|  |  |  |  |  |  | (95% CI) |  | (95% CI) |  |
| **MetS^a^ status** |  |  |  |  | <0.0001 |  | <0.0001 |  | 0.0018 |
| Sustained non-MetS group | 3,907,855 | 13,776 | 0.556 | 0.54(0.52-0.55) |  | 0.85(0.83-0.88) |  | 0.94(0.91-0.97) |  |
| Transition to MetS group | 632,688 | 3,270 | 0.818 | 0.79(0.75-0.82) |  | 0.92(0.88-0.96) |  | 0.95(0.91-0.99) |  |
| Transition to non-MetS group | 502,856 | 2,732 | 0.861 | 0.83(0.79-0.87) |  | 0.94(0.9-0.98) |  | 0.98(0.94-1.03) |  |
| Sustained MetS group | 931,909 | 6,102 | 1.04 | 1 (reference) |  | 1 (reference) |  | 1 (reference) |  |
| **By changes of MetS components** |  |  |  |  |  |  |  |  |  |
| Waist circumference |  |  |  |  | <0.0001 |  | <0.0001 |  | <0.0001 |
| No → No | 3,923,615 | 14,681 | 0.591 | 0.62(0.6-0.64) |  | 0.78(0.75-0.8) |  | 0.83(0.79-0.86) |  |
| No → Yes | 513,925 | 2,317 | 0.713 | 0.75(0.72-0.79) |  | 0.85(0.81-0.89) |  | 0.87(0.83-0.92) |  |
| Yes → No | 444,847 | 2,305 | 0.821 | 0.86(0.82-0.91) |  | 0.89(0.85-0.94) |  | 0.93(0.88-0.97) |  |
| Yes → Yes | 1,092,921 | 6,577 | 0.951 | 1 (reference) |  | 1 (reference) |  | 1 (reference) |  |
| Fasting glucose |  |  |  |  | <0.0001 |  | <0.0001 |  | <0.0001 |
| No → No | 3,359,245 | 11,326 | 0.531 | 0.48(0.47-0.5) |  | 0.77(0.75-0.79) |  | 0.8(0.78-0.83) |  |
| No → Yes | 798,529 | 3,754 | 0.744 | 0.68(0.65-0.7) |  | 0.86(0.83-0.89) |  | 0.87(0.84-0.9) |  |
| Yes → No | 718,981 | 3,206 | 0.705 | 0.64(0.61-0.67) |  | 0.83(0.79-0.86) |  | 0.85(0.81-0.88) |  |
| Yes → Yes | 1,098,553 | 7,594 | 1.103 | 1 (reference) |  | 1 (reference) |  | 1 (reference) |  |
| HDL-cholesterol |  |  |  |  | <0.0001 |  | 0.2159 |  | 0.0143 |
| No → No | 3,906,588 | 15,915 | 0.644 | 0.83(0.8-0.86) |  | 1.03(0.99-1.07) |  | 1.06(1.02-1.1) |  |
| No → Yes | 672,699 | 3,250 | 0.764 | 0.98(0.94-1.03) |  | 1.02(0.98-1.07) |  | 1.03(0.98-1.08) |  |
| Yes → No | 607,832 | 2,828 | 0.736 | 0.95(0.9-0.99) |  | 1.05(1-1.1) |  | 1.06(1.01-1.12) |  |
| Yes → Yes | 788,189 | 3,887 | 0.779 | 1 (reference) |  | 1 (reference) |  | 1 (reference) |  |
| Blood pressure |  |  |  |  | <0.0001 |  | <0.0001 |  | <0.0001 |
| No → No | 2,599,455 | 7,596 | 0.46 | 0.45(0.44-0.46) |  | 0.84(0.81-0.86) |  | 0.89(0.86-0.92) |  |
| No → Yes | 774,397 | 3,179 | 0.649 | 0.63(0.61-0.66) |  | 0.88(0.85-0.92) |  | 0.9(0.87-0.94) |  |
| Yes → No | 657,211 | 2,614 | 0.628 | 0.62(0.59-0.64) |  | 0.88(0.84-0.91) |  | 0.9(0.87-0.94) |  |
| Yes → Yes | 1,944,245 | 12,491 | 1.022 | 1 (reference) |  | 1 (reference) |  | 1 (reference) |  |
| Triglycerides |  |  |  |  | <0.0001 |  | <0.0001 |  | <0.0001 |
| No → No | 3,241,284 | 13,171 | 0.642 | 0.88(0.86-0.91) |  | 1.15(1.12-1.19) |  | 1.28(1.24-1.33) |  |
| No → Yes | 773,869 | 3,508 | 0.716 | 0.98(0.94-1.03) |  | 1.07(1.02-1.11) |  | 1.11(1.07-1.16) |  |
| Yes → No | 663,997 | 3,238 | 0.772 | 1.06(1.02-1.11) |  | 1.1(1.05-1.15) |  | 1.16(1.11-1.21) |  |
| Yes → Yes | 1,296,158 | 5,963 | 0.728 | 1 (reference) |  | 1 (reference) |  | 1 (reference) |  |

**Abbreviations:** HCC, hepatocellular carcinoma; MetS, metabolic syndrome; HR, hazard ratio; CI, confidence interval

^a^Metabolic syndrome and components were defined from blood tests and anthropometric measurements from the 2009−2010 examinations: waist circumference ≥90 cm (male) or 85 cm (female), systolic blood pressure ≥130 mmHg and/or diastolic blood pressure ≥85 mmHg, fasting glucose ≥100 mg/dL, triglycerides ≥150 mg/dL, HDL <40 mg/dL (male) or 50 mg/dL (female). The presence of three or more out of five components was regarded as metabolic syndrome.

^b^Model 1: adjusted for age and sex

^c^Model 2: adjusted age, sex, smoking, alcohol, regular exercise, and BMI

**Supplementary Table 4. Subgroup analysis for the incidence of HCC according to metabolic change during 2 years of follow-up**

|  | No. of subjects | HCC cases (n) | Incidence of HCC  (1,000 person-years) | Crude HR  (95% CI) | Adjusted HR  Model 1  (95% CI) | Adjusted HR  Model 2  (95% CI) |
| --- | --- | --- | --- | --- | --- | --- |
| **Age <65 years** |  |  |  |  |  |  |
| Sustained non-MetS group | 3,583,270 | 10,508 | 0.461 | 1 (reference) | 1 (reference) | 1 (reference) |
| Transition to MetS group | 516,105 | 2,121 | 0.648 | 1.40 (1.34–1.47) | 1.08 (1.03–1.13) | 1.004 (0.96–1.05) |
| Transition to non-MetS group | 404,441 | 1,715 | 0.669 | 1.45 (1.38–1.53) | 1.09 (1.03–1.14) | 1.03 (0.97–1.08) |
| Sustained MetS group | 655,096 | 3,344 | 0.806 | 1.75 (1.68–1.81) | 1.17 (1.13–1.22) | 1.06 (1.01–1.11) |
| **Age ≥65 years** |  |  |  |  |  |  |
| Sustained non-MetS group | 324,585 | 3,268 | 1.636 | 1 (reference) | 1 (reference) | 1 (reference) |
| Transition to MetS group | 116,583 | 1,149 | 1.589 | 0.97 (0.91–1.04) | 1.10 (1.03–1.18) | 1.05 (0.98–1.13) |
| Transition to non-MetS group | 98,415 | 1,017 | 1.675 | 1.02 (0.95–1.10) | 1.16 (1.08–1.24) | 1.12 (1.04–1.20) |
| Sustained MetS group | 276,813 | 2,758 | 1.608 | 0.98 (0.93–1.03) | 1.23 (1.17–1.29) | 1.14 (1.08–1.21) |
| **Male** |  |  |  |  |  |  |
| Sustained non-MetS group | 2,229,163 | 9,483 | 0.673 | 1 (reference) | 1 (reference) | 1 (reference) |
| Transition to MetS group | 389,660 | 2,209 | 0.903 | 1.34 (1.28–1.40) | 1.089(1.04,1.141) | 1.032(0.984,1.083) |
| Transition to non-MetS group | 311,410 | 1,898 | 0.971 | 1.44 (1.37–1.52) | 1.14 (1.08–1.19) | 1.09 (1.03–1.14) |
| Sustained MetS group | 500,751 | 3,810 | 1.219 | 1.81 (1.74–1.88) | 1.25 (1.20–1.29) | 1.15 (1.10–1.20) |
| **Female** |  |  |  |  |  |  |
| Sustained non-MetS group | 1,678,692 | 4,293 | 0.402 | 1 (reference) | 1 (reference) | 1 (reference) |
| Transition to MetS group | 243,028 | 1,061 | 0.685 | 1.7 (1.59–1.82) | 1.12 (1.05–1.20) | 1.02 (0.95–1.09) |
| Transition to non-MetS group | 191,446 | 834 | 0.685 | 1.70 (1.58–1.83) | 1.10 (1.02–1.19) | 1.02 (0.94–1.10) |
| Sustained MetS group | 431,158 | 2,292 | 0.837 | 2.08 (1.97–2.18) | 1.15 (1.09–1.22) | 1.00 (0.94–1.06) |
| **Non-smoker** |  |  |  |  |  |  |
| Sustained non-MetS group | 2,921,053 | 9,730 | 0.525 | 1 (reference) | 1 (reference) | 1 (reference) |
| Transition to MetS group | 463,127 | 2,370 | 0.808 | 1.54 (1.47–1.61) | 1.08 (1.04–1.13) | 1.004 (0.96–1.05) |
| Transition to non-MetS group | 370,179 | 1,953 | 0.835 | 1.59 (1.51–1.67) | 1.09 (1.04–1.15) | 1.03 (0.98–1.08) |
| Sustained MetS group | 722,749 | 4,714 | 1.034 | 1.97 (1.90–2.04) | 1.19 (1.15–1.24) | 1.06 (1.02–1.11) |
| **Smoker** |  |  |  |  |  |  |
| Sustained non-MetS group | 986,802 | 4,046 | 0.650 | 1 (reference) | 1 (reference) | 1 (reference) |
| Transition to MetS group | 169,561 | 900 | 0.846 | 1.30 (1.21–1.40) | 1.08 (1.01–1.16) | 1.05 (0.97–1.13) |
| Transition to non-MetS group | 132,677 | 779 | 0.937 | 1.44 (1.34–1.56) | 1.15 (1.06–1.24) | 1.12 (1.03–1.21) |
| Sustained MetS group | 209,160 | 1,388 | 1.063 | 1.64 (1.54–1.74) | 1.14 (1.08–1.22) | 1.10 (1.03–1.18) |
| **Non- or moderate drinker** |  |  |  |  |  |  |
| Sustained non-MetS group | 3,650,995 | 12,364 | 0.534 | 1 (reference) | 1 (reference) | 1 (reference) |
| Transition to MetS group | 573,871 | 2,842 | 0.784 | 1.47 (1.41–1.53) | 1.07 (1.02–1.11) | 1.00 (0.96–1.04) |
| Transition to non-MetS group | 459,512 | 2,428 | 0.837 | 1.57(1.5–1.64) | 1.11 (1.06–1.16) | 1.05 (1.003–1.10) |
| Sustained MetS group | 852,237 | 5,396 | 1.006 | 1.88 (1.82–1.94) | 1.17 (1.13–1.21) | 1.05 (1.01–1.09) |
| **Heavy drinker** |  |  |  |  |  |  |
| Sustained non-MetS group | 256,860 | 1,412 | 0.870 | 1 (reference) | 1 (reference) | 1 (reference) |
| Transition to MetS group | 58,817 | 428 | 1.157 | 1.33 (1.19–1.48) | 1.10 (0.99–1.23) | 1.16 (1.04–1.30) |
| Transition to non-MetS group | 43,344 | 304 | 1.117 | 1.28 (1.13–1.45) | 1.01 (0.89–1.14) | 1.05 (0.93–1.20) |
| Sustained MetS group | 79,672 | 706 | 1.413 | 1.62 (1.48–1.78) | 1.15 (1.05–1.26) | 1.26 (1.14–1.40) |
| **Regular physical activity (–)** |  |  |  |  |  |  |
| Sustained non-MetS group | 3,127,323 | 10,804 | 0.545 | 1 (reference) | 1 (reference) | 1 (reference) |
| Transition to MetS group | 507,756 | 2,565 | 0.800 | 1.47 (1.40–1.53) | 1.06 (1.02–1.11) | 1.00 (0.951,1.04) |
| Transition to non-MetS group | 390,819 | 2,151 | 0.874 | 1.6 (1.53–1.68) | 1.12 (1.07–1.17) | 1.06 (1.01–1.11) |
| Sustained MetS group | 742,126 | 4,821 | 1.033 | 1.89 (1.83–1.95) | 1.17 (1.13–1.21) | 1.06(1.02–1.1) |
| **Regular physical activity (+)** |  |  |  |  |  |  |
| Sustained non-MetS group | 780,532 | 2,972 | 0.599 | 1 (reference) | 1 (reference) | 1 (reference) |
| Transition to MetS group | 124,932 | 705 | 0.891 | 1.49 (1.37–1.62) | 1.14 (1.05–1.24) | 1.07 (0.99–1.17) |
| Transition to non-MetS group | 112,037 | 581 | 0.819 | 1.37 (1.25–1.50) | 1.05 (0.96–1.15) | 1.00 (0.91–1.10) |
| Sustained MetS group | 189,783 | 1,281 | 1.069 | 1.78 (1.67–1.91) | 1.20 (1.13–1.29) | 1.10 (1.02–1.19) |

**Abbreviations:** HCC, hepatocellular carcinoma; MetS, metabolic syndrome; HR, hazard ratio; CI, confidence interval

Model 1: adjusted for age and sex

Model 2: adjusted age, sex, smoking, alcohol, regular exercise, and BMI

**Supplementary Table 5. Stratified analysis for the incidence of HCC according to age subgroup**

|  | No. of subjects | HCC cases (n) | Incidence of HCC | Crude HR | Adjusted HR | Adjusted HR |
| --- | --- | --- | --- | --- | --- | --- |
|  |  |  | (1,000 person-years) | (95% CI) | Model 1 | Model 2 |
|  |  |  |  |  | (95% CI) | (95% CI) |
| **Age <45 years** |  |  |  |  |  |  |
| Sustained non-MetS group | 1,969,558 | 1,649 | 0.13 | 1 (reference) | 1 (reference) | 1 (reference) |
| Transition to MetS group | 191,025 | 207 | 0.173 | 1.33 (1.04-1.71) | 1.02 (0.80-1.31) | 0.92 (0.71-1.19) |
| Transition to non-MetS group | 144,737 | 192 | 0.211 | 1.63 (1.26-2.11) | 1.21 (0.93-1.56) | 1.11 (0.85-1.45) |
| Sustained MetS group | 176,301 | 249 | 0.226 | 1.74 (1.38-2.19) | 1.18 (0.94-1.49) | 1.00 (0.77-1.30) |
| **Age 45-55 years** |  |  |  |  |  |  |
| Sustained non-MetS group | 1,108,132 | 3,448 | 0.493 | 1 (reference) | 1 (reference) | 1 (reference) |
| Transition to MetS group | 177,328 | 696 | 0.623 | 1.26 (1.1-1.46) | 1.07 (0.93-1.23) | 0.99 (0.86-1.14) |
| Transition to non-MetS group | 134,770 | 462 | 0.545 | 1.10 (0.94-1.31) | 0.91 (0.77-1.08) | 0.85 (0.72-1.01) |
| Sustained MetS group | 233,623 | 1,009 | 0.687 | 1.39 (1.24-1.58) | 1.09 (0.96-1.23) | 0.95 (0.83-1.10) |
| **Age >55 years** |  |  |  |  |  |  |
| Sustained non-MetS group | 830,165 | 8,679 | 1.617 | 1 (reference) | 1 (reference) | 1 (reference) |
| Transition to MetS group | 264,335 | 2,367 | 1.498 | 0.93 (0.85-1.08) | 1.09 (1.00-1.18) | 1.01 (0.93-1.10) |
| Transition to non-MetS group | 223,349 | 2,078 | 1.718 | 1.06 (0.97-1.14) | 1.15 (1.06-1.25) | 1.08 (0.99-1.17) |
| Sustained MetS group | 521,985 | 4,844 | 1.458 | 0.90 (0.79-1.07) | 1.32 (1.24-1.40) | 1.18 (1.10-1.26) |

**Abbreviations:** HCC, hepatocellular carcinoma; MetS, metabolic syndrome; HR, hazard ratio; CI, confidence interval

Model 1: adjusted for age and sex

Model 2: adjusted age, sex, smoking, alcohol, regular exercise, and BMI
